# Improved Municipal Sanitation

**Published:** 1907-01

**Authors:** 


**A Monthly Review of Medicine and Surgery.**

*EDITOR:*

WILLIAM WARREN POTTER, M. D.

All communications, whether of a literary or business nature, books for review and exchanges, should be addressed to the editor 284 FRANKLIN ST., BUFFALO, N. Y.

VOL. LXII. JANUARY, 1907. No. 6

### Improved Municipal Sanitation.

SEVERAL questions formerly irrelevant to the problems of municipal sanitation are now becoming paramount. Great progress has been made in the control of communicable and preventable diseases and the annual death rate has been greatly reduced through the efficiency of the health officers in this respect, not only in Buffalo but also in many other large cities.

The time seems to be appropriate to take up collateral questions relating to the health and comfort of our citizens. Elsewhere, in this issue, we publish an important communication from Dr. Dewitt C. Greene, entitled, "The Air we Breathe," which deals intelligently with the smoke nuisance,—an ever increasing detriment to the health and comfort of this community. In New York it is unlawful to burn soft coal in factories, hotels, homes or apartment houses, and Dr. Darlington, the health commissioner, has instituted prosecutions with determined vigor against violators of the law and has obtained in each case a conviction that has served to prevent further infraction. It would be well if the health commissioner of Buffalo were given similar powers.

Another important factor causing distress and perhaps danger to the sick, which seems timely for the health department to deal with is the unnecessary noises incident to the traffic and pleasure of large cities. This problem has already been handled successfully in many cities but as yet Buffalo has not outgrown its swaddling clothes in this respect and perhaps in some others; at all events, it behaves very much like an overgrown village with reference to many problems relating to the preservation of health, and the prevention of disease, as well as those relating to the increase of its business facilities.

In New York, again, we find an example worth heeding. A crusade has been organised by a new society for the suppression of unnecessary noises, led by Mrs. Isaac L. Rice. This philanthropic, high minded and progressive woman already has successfully conducted a crusade against whistling tugs in the harbor. The hideous whistles have been put out of commission. In a recent interview Mrs. Rice outlined her plans and hopes, indicating that she has begun a long-needed crusade against the noises which make life a hideous bedlam for those confined in the hospitals and public institutions. She said in part:

I have long felt that there has been an urgent need for such a society, but as nobody else appeared willing to take the initiative in the matter I have finally had to do so myself. The noise question grows more important every year in this city, and it has become more aggravated of late than some people imagine. It is an evil which in most cases is entirely unnecessary and therefore the more objectionable. This is not an anti-noise society, but is only against unnecessary noises.

One of the things that the new society will ask the city administration's aid in, will be the detailing of a policeman on each hospital beat to prevent the various unpleasant sounds that are at present made by the small boys of the surrounding neighborhood. One of their customs is to get on their little go-carts at the top of a street on a certain hospital block, then ride down close to the fence and scrape a stick along the railing, producing an ear splitting noise which is especially annoying to the sick.

As an instance of how it would be possible to quell the various needless noises about the hospitals, Mrs. Rice mentioned the fact that St. Vincent's Hospital, in Seventh avenue, between 11th and 12th streets, which had been erected before he tracks of the street railway were laid in that avenue, had entered into an agreement with the railway authorities at that time never to ring their bells when passing the institution. This rule has been rigidly adhered to.

The milk wagon nuisance was touched on by Mrs. Rice. "Our society will endeavor to have an ordinance passed which will make it compulsory for all business vehicles, such as milk and bakers' wagons, which have to be on the streets before the hour of 6 in the morning, to be equipped with rubber tires, and to fix the milk cans in such a manner that the noise created by the shaking of the wagon will be done away with," said Mrs. Rice. "I for one will refuse to trade with a dealer who does not comply with this rule."

Factory whistles and sirens on river craft on the North and East rivers will also be made objects of crusade.

There is no possible argument that can logically prevail against the proposition that unnecessary noises prolong ill health, increase the severity of many diseases, and should be suppressed in the interest of preventive as well as curative medicine.

In regard to spitting on the sidewalks there is but one view to take, which is, that it should be suppressed. It is filthy, disease-breeding, and ungentlemanly. In many cities a fine is imposed for each offense and in some the enforcement is so rigid that visitors are warned by their friends or acquaintances beforehand, lest they are caught unawares in the toils of the law. It is a great pity that similar ordinances are not enforced in Buffalo.

